# Supplementary material for: Endoglin as a BMP9 Co-Receptor in Vascular Endothelial Cells: Prodomain Displacement and TGFBRII Recruitment
Source: Nat Commun. Author manuscript; Available in PMC 2026 Feb 14. (PMC12824264; doi:10.1038/s41467-025-67531-9)
Supplement: Supplementary Information [file EMS211989-supplement-Supplementary_Information.zip › 41467_2025_67531_MOESM3_ESM.pdf]

# Description of Additional Supplementary Files

**Supplementary Data 1:** Detailed information on antibodies and qPCR primers used in this study.
