# Supplementary material for: Endoglin as a BMP9 Co-Receptor in Vascular Endothelial Cells: Prodomain Displacement and TGFBRII Recruitment
Source: Nat Commun. Author manuscript; Available in PMC 2026 Feb 14. (PMC12824264; doi:10.1038/s41467-025-67531-9)
Supplement: Supplementary Information [file EMS211989-supplement-Supplementary_Information.zip › 41467_2025_67531_MOESM1_ESM.pdf]

SUPPLEMENTARY INFORMATION

Endoglin as a BMP9 Co-Receptor in Vascular Endothelial Cells: Prodomain Displacement and TGFBRII Recruitment

Jingxu Guo[1], Karolina Kostrzyńska[1], Ioannis Kamzolas[2,3] Xudong Yang[1], Midory Thorikay[4], Eckart De Bie[1], Rowena. J. Jones[1], Adam Brownstein[5], Lu Long[1], Christopher J. Rhodes[6], Allan Lawrie[6], Martin R. Wilkins[6], Esmee Groeneveld[4], Zhen Tong[1], Marie-José Goumans[4], Evangelia Petsalaki[3], Jason Hong[5], Mark R. Toshner[1], Antonio Vidal-Puig[1,2,7], Helen M. Arthur[8], Wei Li[1,*]

1. The Victor Phillip Dahdaleh Heart & Lung Research Institute, School of Clinical Medicine, University of Cambridge. Papworth Road, Cambridge Biomedical Campus, Cambridge, CB2 0BB, United Kingdom
2. Institute of Metabolic Science, MRC Metabolic Diseases Unit, University of Cambridge. Box 289, Addenbrooke's Hospital, Cambridge, CB2 0QQ, United Kingdom
3. European Molecular Biology Laboratory, European Bioinformatics Institute (EMBL-EBI), Wellcome Genome Campus, Hinxton, Cambridge, CB10 1SD, United Kingdom
4. Department of Cell and Chemical Biology, Leiden University Medical Centre, Leiden, 2300 RC, The Netherlands
5. Division of Pulmonary and Critical Care Medicine, University of California, Los Angeles, United States
6. National Heart and Lung Institute, Imperial College London, London, United Kingdom
7. Centro de Investigacion Principe Felipe, 46012 Valencia, Spain.
8. Biosciences Institute, International Centre for Life, Newcastle University, Newcastle upon Tyne, NE1 3BZ, United Kingdom

TABLE OF CONTENTS:

I. Supplementary Methods

ELISA-based binding assay

Surface plasmon resonance analysis

II. Supplementary Table 1. Description of statistical parameters for Figure 9.

III. Supplementary Figures and Figure Legends

Supplementary Figure 1. Prodomains, type II receptors and ENG bind to BMP9 and BMP10 at overlapping sites .

Supplementary Figure 2. Isoelectric points of proteins and complexes and additional data supporting BMP9 native PAGE in Figure 1a.

Supplementary Figure 3. Additional data supporting native PAGE in Figure 1d, 1g and 1h.

Supplementary Figure 4. ALK1 binding does not affect the affinity of BMP9 and BMP10 for ENG.

Supplementary Figure 5. TGFBRII ECD does not bind to BMP9 GFD.

Supplementary Figure 6. LC-MS/MS sequence coverage of ENG and the binding partners ALK1, SMAD1, SMAD2 and SMAD5 in the experiment described in Figure 5d.

Supplementary Figure 7. Additional data supporting the model in Figure 6.

Supplementary Figure 8. Evaluating ENG knockout efficiency by immunofluorescence staining and RT-qPCR

Supplementary Figure 9. Pathway analysis of DEGs from Figure 7h.

Supplementary Figure 10. Structural analysis showing ENG, TGFBRII and ALK1 can interact with BMP9 without clash

Supplementary Figure 11. *TGFBR2* is highly expressed in vascular endothelial cells.

IV. Supplementary References

I. Supplementary Methods:

ELISA-based binding assay

A high binding 96-well ELISA plate was coated with in-house purified monomeric receptor extracellular domains (ECDs) of BMPRII, ActRIIB, TGFBRII and ENG(M) at 200 nM, or ENG(D) at 100 nM, or BMPRII-Fc, ActRIIB-Fc and TGFBRII-Fc (dimeric receptor ECDs) at 5 nM (all Fc-fusion proteins were from R&D Systems) in PBS at 4 °C overnight. After washing three times with PBS, wells were blocked with 1%BSA/PBS for 2 hours and then washed three times with 0.05%Tween/PBS. BMP9 GFD (100 µl) was added in duplicate to the wells at 206, 103, 51.5, 25.8, 12.9, 6.4 and 0 pM, and incubated at RT for 2 hours. Wells were again washed three times with 0.05% Tween-20/PBS and then probed with biotinylated anti-BMP9 antibody (at 0.8 µg/ml; BAF3209) at RT for 2 hours. After washing three times with 0.05% Tween-20/PBS, ExtraAvidin-Alkaline Phosphatase conjugate (Sigma Aldrich E2636, 1:400 in 1%BSA/PBS) was added and incubated for a further 2 hours. Wells were washed twice with 0.05%Tween/PBS and followed by wash with water before pNPP solution (Sigma Aldrich N2640) was added. Development was carried out in the dark at room temperature, with readings taken every 10 minutes at 405 nm on a Bio-Rad 680 microplate reader until the signals were fully developed.

Surface plasmon resonance analysis

Receptor-ligand binding experiments in **Supplementary Figure 5a, b** were performed using a Biacore T100 biosensor (Biacore/GE Healthcare). In-house purified receptor ECDs (ActRIIB, TGFBRII and BMPRII) were immobilised onto a Series S research grade CM5 sensor chip by amine-coupling with a targeted immobilisation level of 1000 RU. For kinetic measurements, a series of BMP9 GFD or TGF-β3 (R&D systems) were injected in duplicate over the flow cells at a flow rate of 30 µl/min in a buffer containing 0.01 M HEPES, 0.5 M NaCl, 3 mM EDTA, 0.5 mg/ml BSA and 0.01% (v/v) Surfactant P20 (pH 7.4) at 25 °C. The binding surface was regenerated between each cycle using 2.5 M Guanidine hydrochloride. The kinetic rate constants were obtained by fitting the corrected data to a 1:1 interaction model using BiaEvaluation software (GE Healthcare) or determined with steady-state analysis. The equilibrium dissociation constant K_D was determined by the ratio of binding rate constants k_{off}/k_{on} or by steady-state kinetics fit.

II. Supplementary Table 1. Description of statistical parameters for Figure 9.

Supplementary Table 1A. Expression values for *ENG* (GSE113439), *NOG* (GSE113439) and *ADAMTSL2* (GSE113439) in lung measured by microarray (Log2 expression) for controls (n=11) and PAH (n=15). Data shown as median(Interquartile range, IQR). Statistical test shown as Mann-Whitney.

	Control	PAH	P value
ENG	11.32(0.5)	10.86(0.4)	0.001
NOG	6.795(0.903)	6.341(0.327)	0.0037
ADAMTSL2	9.077(0.399)	8.628(0.318)	<0.0001

Supplementary Table 1B. Expression values for *ENG* and *NOG* in whole blood measured by RNAseq (Transcripts Per Million, TPM) for controls (n=126) and PAH (n=363). Data shown as median(IQR). Statistical test shown as Mann-Whitney.

	Control	PAH	P value
ENG	0.974(0.534)	0.866(0.519)	0.0052
NOG	7.113(5.841)	4.382(4.856)	<0.0001

Supplementary Table 1C. Proteomic expression values for soluble Endoglin and Noggin in plasma proteome measured by SomaScan for controls (n=108) and PAH (n=463). Values are Z-value difference in PAH versus healthy controls. Data shown as median(IQR). Statistical test shown as Mann-Whitney.

	Control	PAH	P value
sEndoglin	-0.0665(1.343)	0.745(1.547)	<0.0001
Noggin	-0.205(1.327)	-0.453(1.166)	0.0014

III. Supplementary Figures and Figure Legends:

Supplementary Figure 1

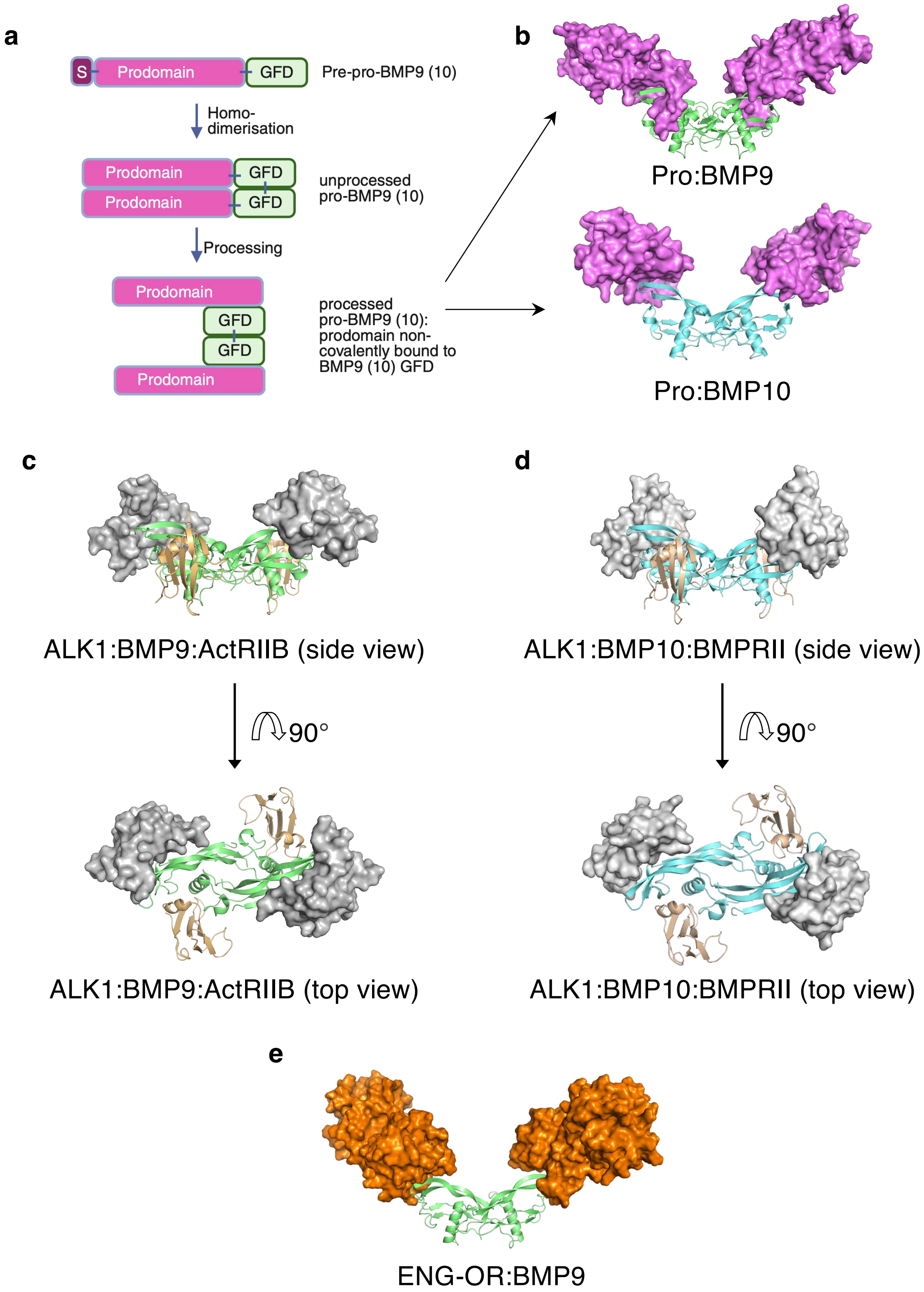

Supplementary Figure 1. Prodomains, type II receptors and ENG bind to BMP9 and BMP10 at overlapping sites. **a** Synthesis and processing of BMP9 or BMP10. S=signal peptide. GFD=growth factor domain. **b** Crystal structures of pro:BMP9 (pdb ID: 4YCG) and pro:BMP10 (pdb ID: 7POI, prodomain is truncated in this structure). **c** The crystal structure of ternary signalling complex ALK1:BMP9:ActRIIB in side and top views (pdb ID: 4FAO). **d** The crystal structure of ternary signalling complex ALK1:BMP10:BMPRII in side and top views (pdb ID: 7PPC). **e** The crystal structure of ENG orphan domain (ENG-OR) in complex with BMP9 (pdb ID: 5HZW). In **b** to **e**, BMP9 and BMP10 GFDs are shown in green and cyan cartoon, respectively. ALK1 is shown in wheat-coloured cartoon. Prodomain, type II receptors and ENG-OR are shown as surface in purple violet, grey and orange, respectively. Panel **a** was generated using BioRender. Structural figures were prepared using Pymol (The PyMOL Molecular Graphics System, Version 2.0, Schrödinger).

Supplementary Figure 2

a

Protein	Residues	MW (kDa)	Isoelectric point (pI)
sENG-6His	26-586+6His	61.6	6.19
BMP9	320-429	12.1	6.85
Pro:BMP9	23-429	45.1	6.03
BMP9 prodomain	23-319	33.1	5.79
BMP10	317-424	12.2	8.67
Pro:BMP10	22-424	45.9	4.89
BMP10 prodomain	22-316	33.8	4.54
BMPRII ECD	27-150	13.9	4.89
ActRIIB ECD	24-115	10.9	4.74

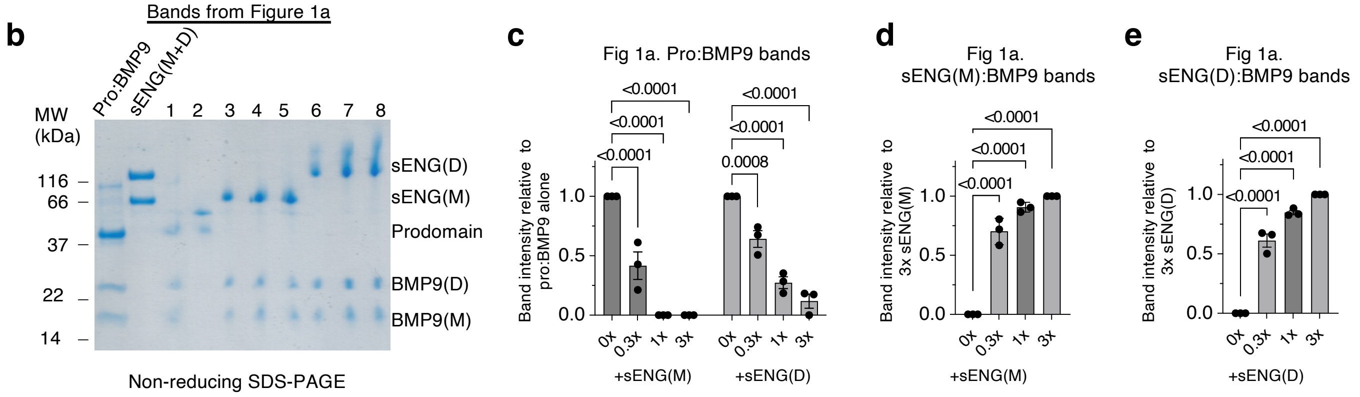

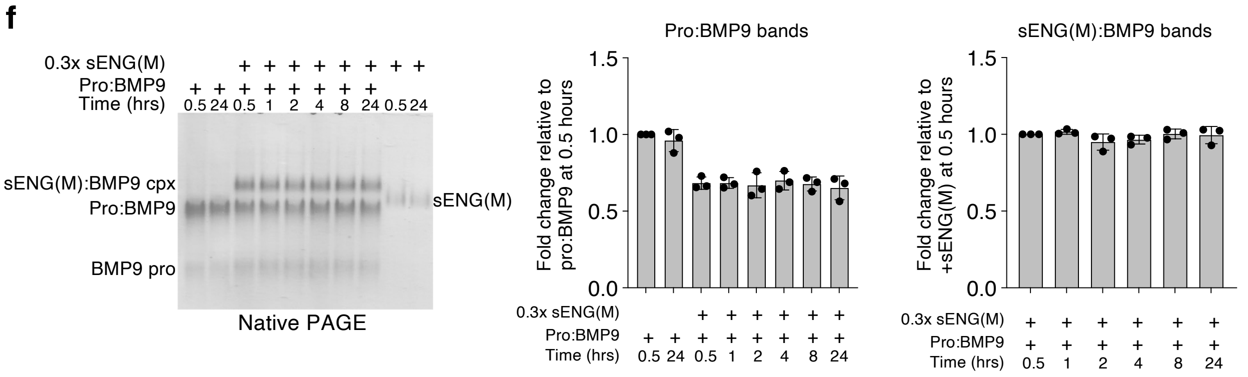

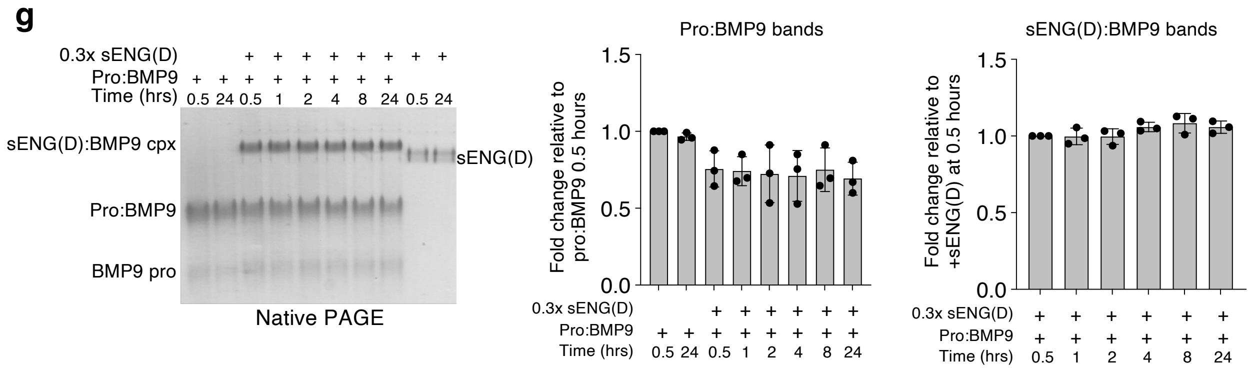

Supplementary Figure 2. Isoelectric point of proteins and complexes and additional data supporting BMP9 native PAGE in Figure 1a. a Residue range, molecular weight (MW) and calculated isoelectric points (pI) of recombinant proteins and protein complexes used in this study. **b** Identification of the bands on the native PAGE in **Figure 1a**. Bands 1-8 in **Figure 1a** were cut out and run on a 12% non-reducing SDS-PAGE, alongside pro:BMP9 and sENG(M+D) as controls to confirm their identity. **c-e** Quantification of bands in Figure 1**a**. **f, g** Time course BMP9 prodomain displacement assays using 0.3-fold excess of sENG(M) (**f**) and sENG(D) (**g**) with densitometry quantification of the complex bands to the right. N=3 independent repeats (**c, d, e, f, g**). Means ± SEM are shown (**c, d**, **e, f, g**). Two-way ANOVA (**c**) or one-way ANOVA (**d, e**) were used with Dunnett's post-tests again relative controls, with only significant p-values (<0.05) shown.

Supplementary Figure 3

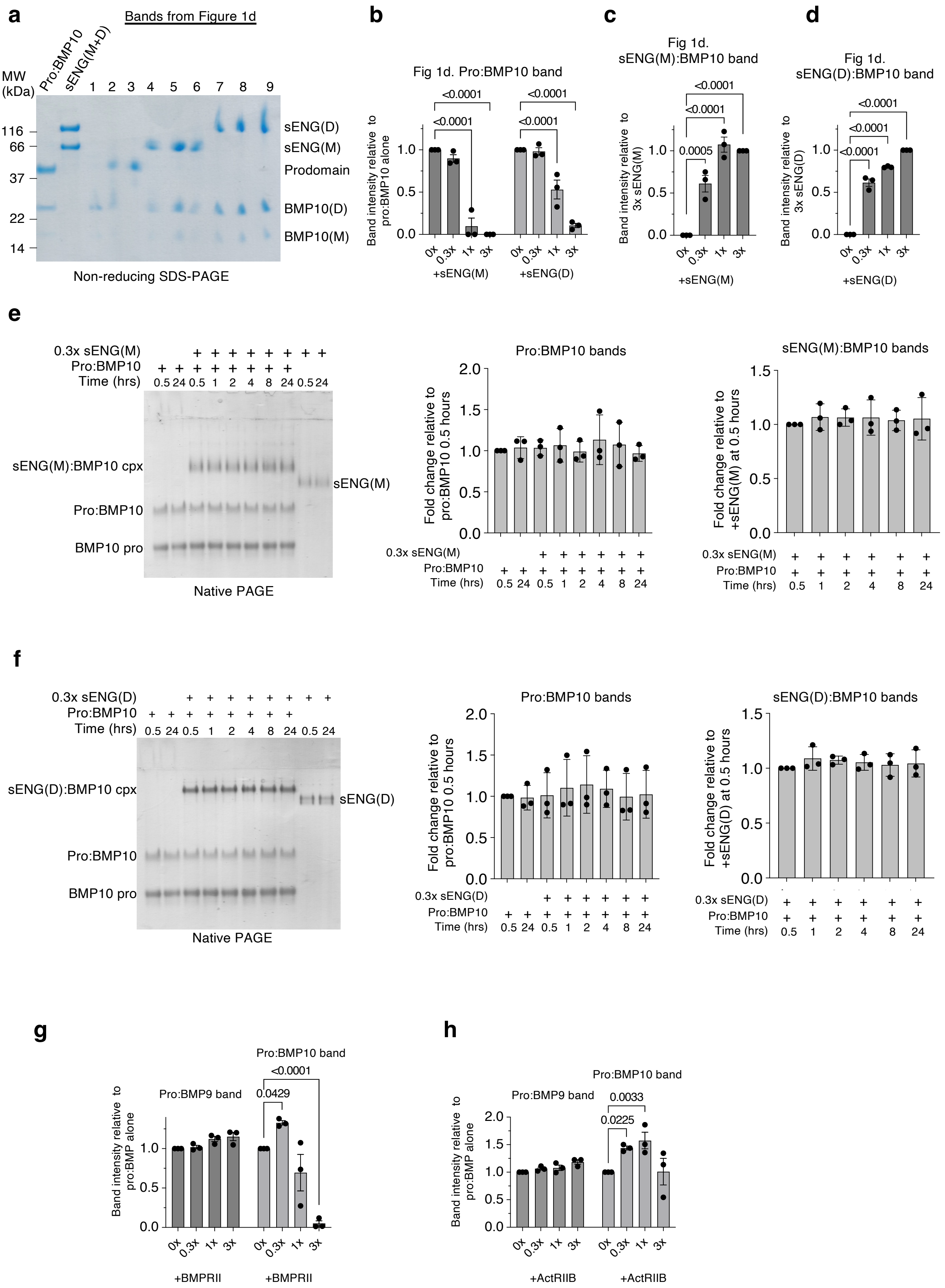

Supplementary Figure 3. Additional data supporting native PAGE in Figure 1d, 1g and 1h. a Identification of the bands on the native PAGE in **Figure 1d**. Bands 1-9 in **Figure 1d** were cut out and run on a 12% non-reducing SDS-PAGE, alongside pro:BMP10 and sENG(M+D) as controls to confirm their identity. **b-d**, Quantification of bands in **Figure 1d**. **e, f** Time course BMP10 prodomain displacement assays using 0.3-fold excess of sENG(M) (**e**) and sENG(D) (**f**) with densitometry quantification of the complex bands to the right. Note that in **b, e** and **f** quantification of pro:BMP10 bands, there was no decrease after addition of 0.3x sENG. This was because pro:BMP10 was already partially dissociated on the native PAGE as demonstrated with a weak pro:BMP10 band and a strong prodomain band. The BMP10 GFD is positively charged therefore retained in the loading well/stacking gel. The 0.3x sENG has complexed with those BMP10 GFD as evidenced by the appearance of the sENG:BMP10 bands, hence no further reduction of pro:BMP10 band was observed. **g** Quantification of bands in **Figure 1g**. **h** Quantification of bands in **Figure 1h**. N=3 independent experiments (**b, c, d, e, f, g, h**). Means ± SEM are shown (**b, c, d, e, f, g, h**). Two-way ANOVA (**b, g, h**) or one-way ANOVA (**c, d**) were used with Dunnett's post-tests again relative controls. Only significant p-values (<0.05) are shown.

Supplementary Figure 4

a

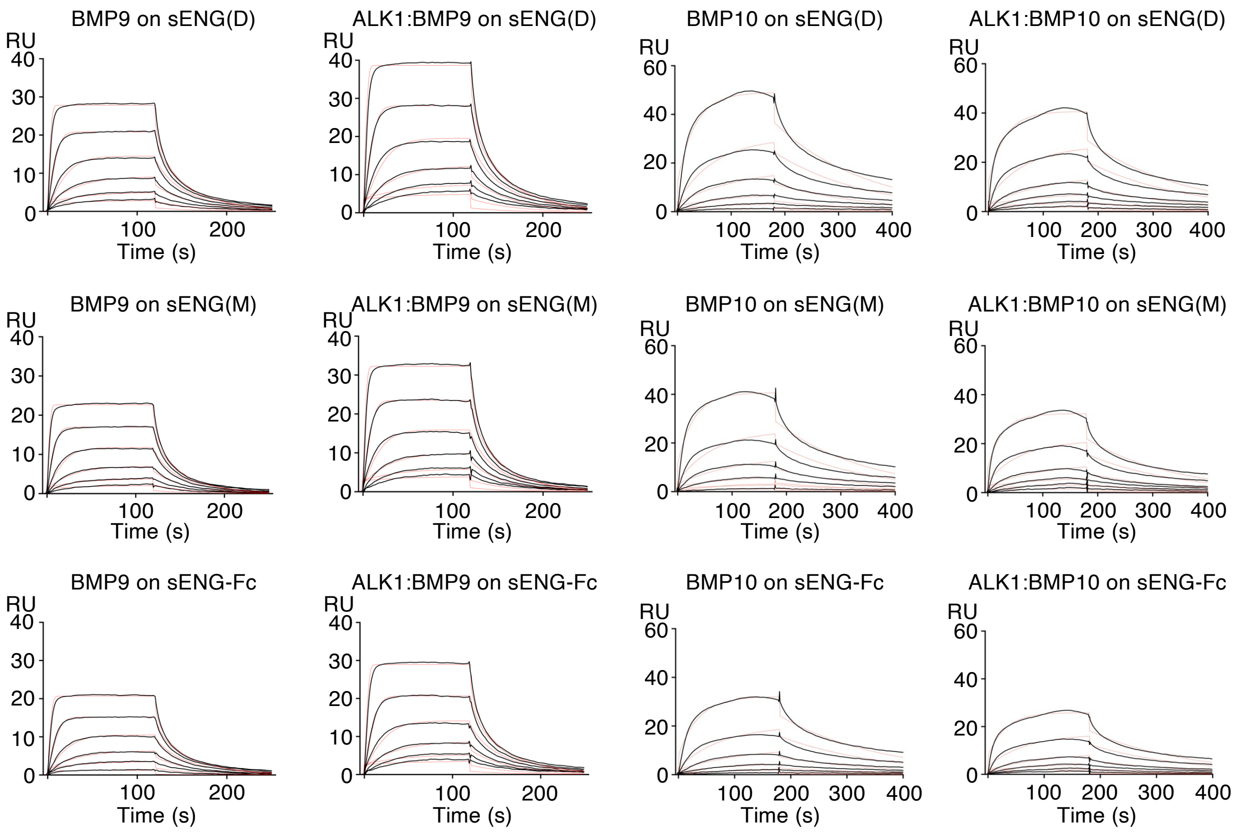

b

	k_{on} ($M^{-1}s^{-1}$)	k_{off} (s^{-1})	K_D (M)
BMP9			
sENG(D)	5.68×10^{7}	1.33×10^{-1}	2.34×10^{-9}
sENG(M)	5.80×10^{7}	1.41×10^{-1}	2.45×10^{-9}
sENG-Fc	5.70×10^{7}	1.50×10^{-1}	2.64×10^{-9}
ALK1:BMP9			
sENG(D)	5.33×10^{7}	1.21×10^{-1}	2.28×10^{-9}
sENG(M)	5.67×10^{7}	1.42×10^{-1}	2.50×10^{-9}
sENG-Fc	6.03×10^{7}	1.58×10^{-1}	2.63×10^{-9}
BMP10			
sENG(D)	1.16×10^{6}	5.85×10^{-3}	5.23×10^{-9}
sENG(M)	1.29×10^{6}	6.21×10^{-3}	4.58×10^{-9}
sENG-Fc	1.06×10^{6}	5.81×10^{-3}	5.46×10^{-9}
ALK1:BMP10			
sENG(D)	1.28×10^{6}	5.69×10^{-3}	4.46×10^{-9}
sENG(M)	1.46×10^{6}	6.12×10^{-3}	4.21×10^{-9}
sENG-Fc	1.26×10^{6}	5.94×10^{-3}	4.73×10^{-9}

Supplementary Figure 4. ALK1 binding does not affect the affinity of BMP9 and BMP10 for ENG. a SPR sensorgrams of BMP9 and BMP10 binding to sENG(M), sENG(D) and sENG-Fc in the absence and presence of ALK1 (at 1.2x molar ratio to BMP GFD dimer). BMP9 and BMP10 were preincubated with ALK1 for 30 min at room temperature before applying to the flow cells. Both BMP9 and BMP10 were applied from 22.22 nM to 0.09 nM in 3-fold serial dilution. **b** Summary of the binding kinetics. All measurements are the averages from two runs.

Supplementary Figure 5

a

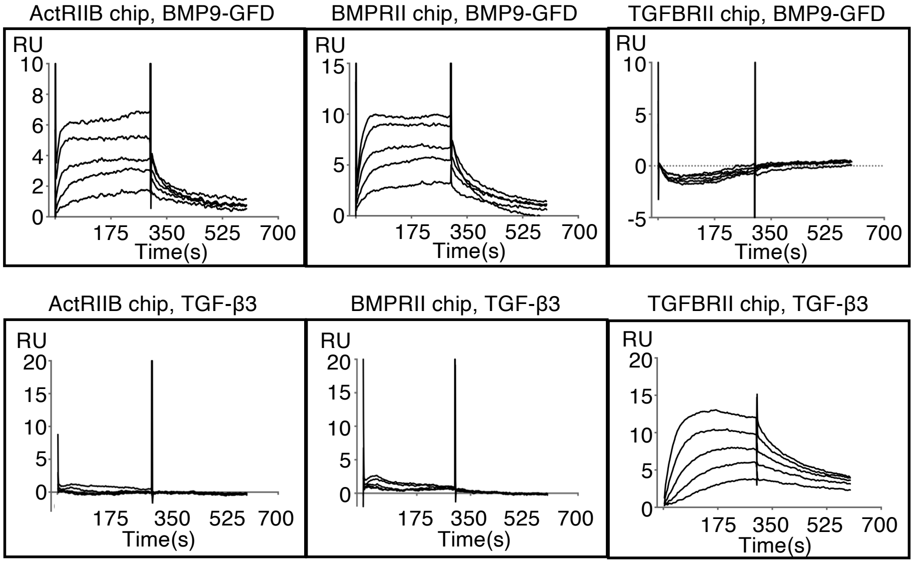

b

Immobilised ECD	Flow ligand	K_D (M)	K_D^* (M)
ActRIIB	BMP9-GFD	8.23 x 10^{-09}	1.18 x 10^{-09}
ActRIIB	TGF-β3	ND	ND
BMPRII	BMP9-GFD	3.07 x 10^{-09}	1.89 x 10^{-09}
BMPRII	TGF-β3	ND	ND
TGFBRII	BMP9-GFD	ND	ND
TGFBRII	TGF-β3	6.64 x 10^{-09}	N/A

* K_D value obtained from steady-state fitting

c

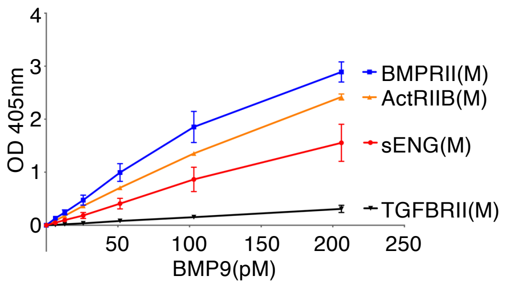

d

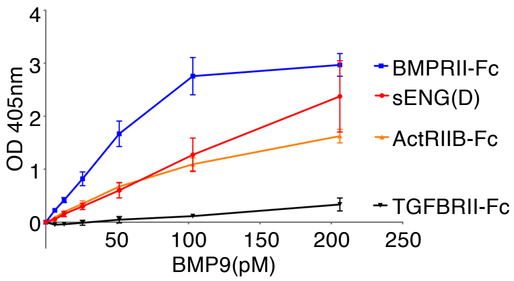

Supplementary Figure 5. TGFBRII ECD does not bind to BMP9 GFD. a SPR sensorgrams showing BMP9 GFD and TGF-β3 binding to chips coated with ActRIIB-Fc, BMPRII-Fc and TGFBRII-Fc, respectively. Although BMP9 GFD can bind to BMPRII-Fc and ActRIIB-Fc in our assay condition, we could not detect BMP9 binding to TGFBRII-Fc. Control experiment showing TGFBRII-Fc coated surface is functional because it can bind TGF-β3 effectively. **b** Summary of the binding kinetics. **c, d** ELISA binding results of BMP9 GFD binding to monomeric type II receptor ECDs and ENG(M), as well as to BMPRII-Fc, ActRIIB-Fc and ENG(D). We did not detect BMP9 GFD binding to either monomeric TGFBRII ECD or TGFBRII-Fc.

Supplementary Figure 6

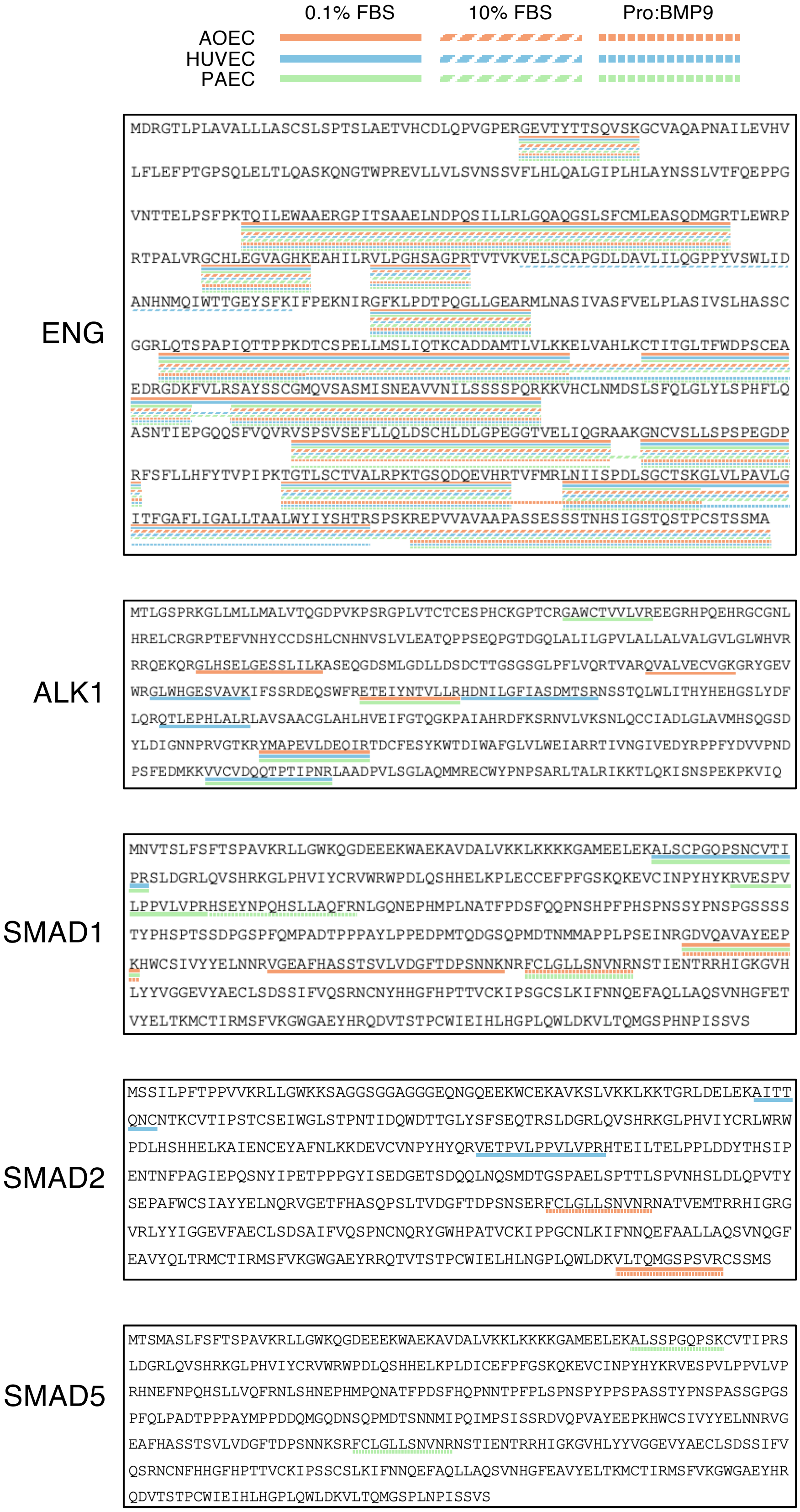

Supplementary Figure 6. LC-MS/MS sequence coverage of ENG and the binding partners ALK1, SMAD1, SMAD2 and SMAD5 in the experiment described in Figure 5d. Peptides detected in the mass spectrometry are highlighted under the sequences of different proteins.

Supplementary Figure 7

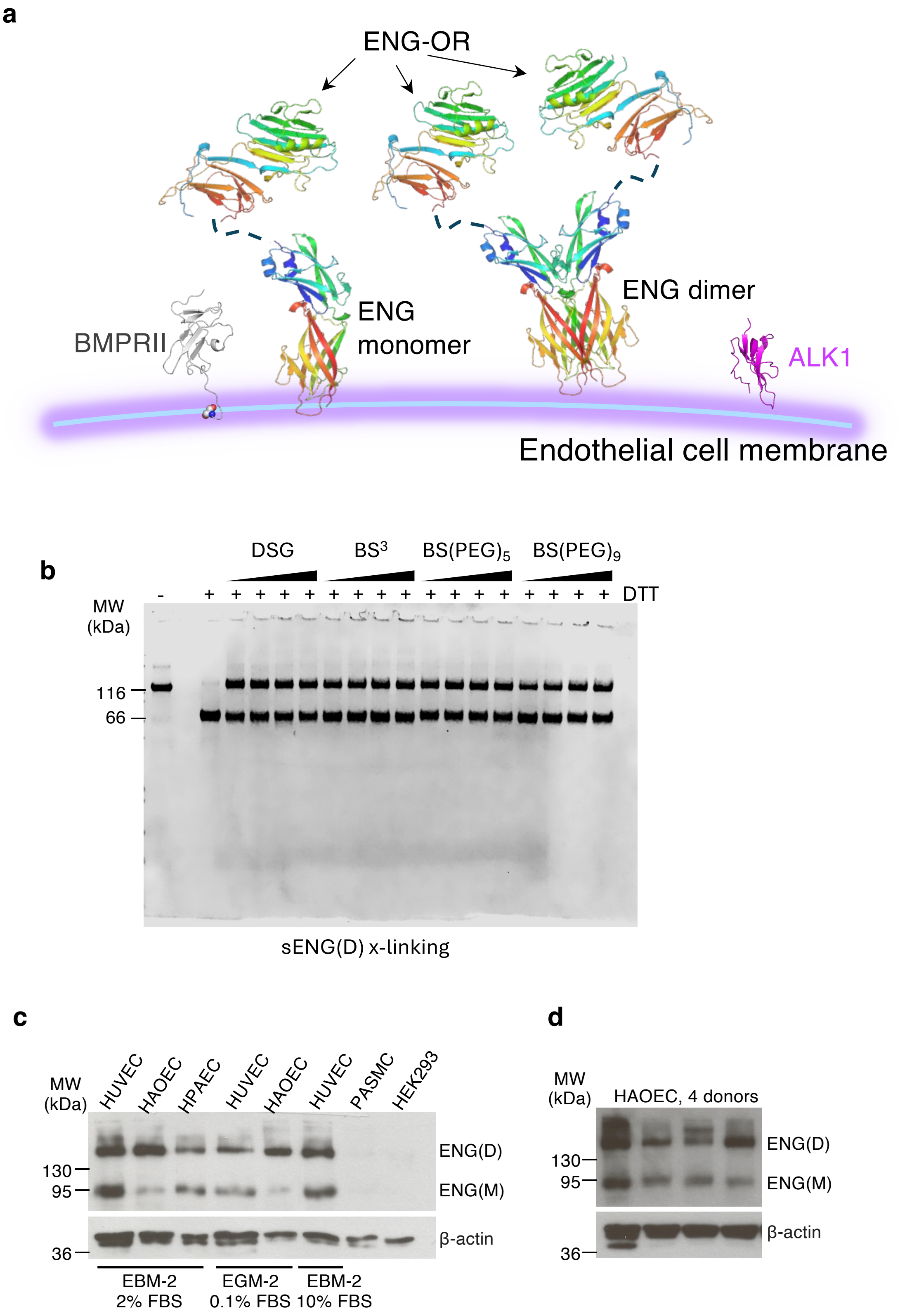

Supplementary Figure 7. Additional data supporting the model in Figure 6. a Structures and models comparing the sizes of extracellular domains of BMPRII, ALK1 and ENG. Only the extracellular parts of the proteins are shown. BMPRII and ALK1 ECDs were taken from the structure of ALK1:BMP10:BMPRII ternary complex (pdb ID: 7PPC)[1]. ENG orphan domain (ENG-OR) and ENG ZP domain were taken from structures with pdb IDs 5HZW and 5HZV, respectively[2]. Full-length ENG extracellular domain was built by connecting ENG-OR to the ZP domain with a dashed line which represents the 19 amino acids-linker between ENG-OR and the ZP domain[2]. Models for both dimeric and monomeric ENG are shown and coloured in rainbow. BMPRII is shown in grey and ALK1 shown in magenta. Structural figures were prepared using Pymol. **b** sENG(D) crosslinking experiment with increasing concentrations of crosslinkers. For each crosslinker, four concentrations were tested (final concentrations): 0.25, 1, 3 and 6 mM. The concentration of sENG(D) in the experiment was 1.15 μM. **c, d** Immunoblots from non-reducing SDS-PAGE of whole cell lysates. Both ENG dimer and monomer were detected in human primary ECs from different vascular beds and cultured under different conditions (**c**) and from different donors of HAOECs (**d**).

Supplementary Figure 8

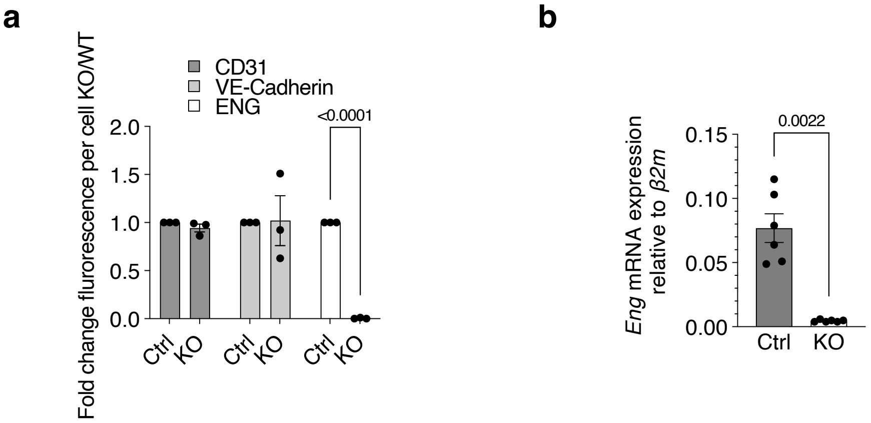

Supplementary Figure 8. Evaluating ENG knockout efficiency by immunofluorescence staining and RT-qPCR. **a** Quantification of immunofluorescence intensity in **Figure 7c**. N=3 biological repeats. Fluorescence images were acquired from cells isolated from three different mice. For each stained cell line, 4–5 images were taken from distinct regions for each protein and average intensity per cell (IntDen/cell) was quantified using Fiji software. **b** *Eng* mRNA expression relative to *β2m* using the ΔCT method. N=6 biological repeats. Means ± SEM are shown (**a**, **b**). Two-way ANOVA, with Dunnett's post-tests between Ctrl and KO for each marker (a) or two-tailed Mann-Whitney test (b) was applied. Only significant p-values (<0.05) are shown.

Supplementary Figure 9

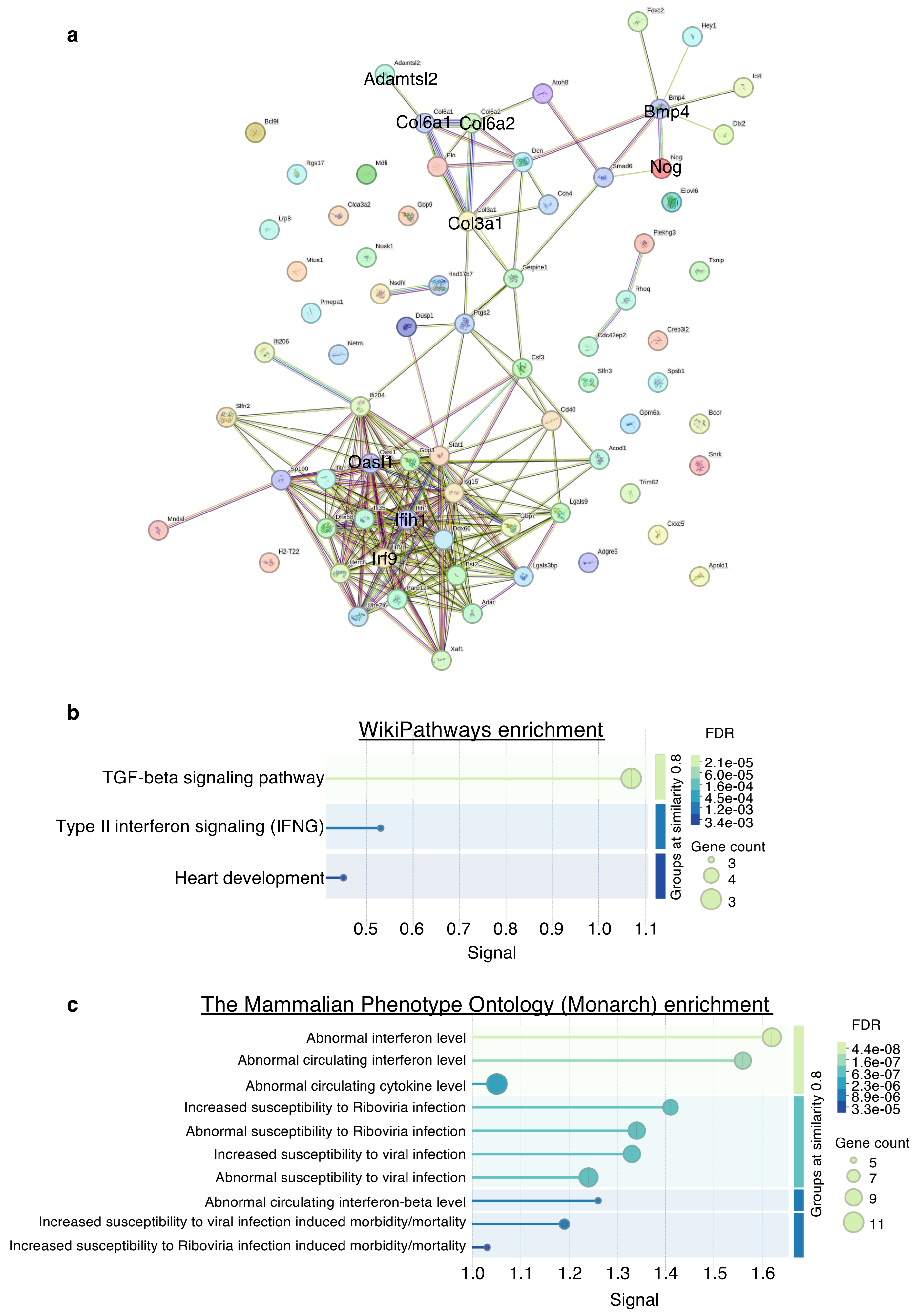

Supplementary Figure 9. Pathway analysis of DEGs from Figure 7h. a Interaction network generated using STRING (https://string-db.org/). Key genes mentioned in the text are highlighted with larger text. **b, c** Significantly enriched Wikipathways (**b**) and Mammalian phenotype Ontology (Monach) (**c**) revealed by String analyses.

Supplementary Figure 10

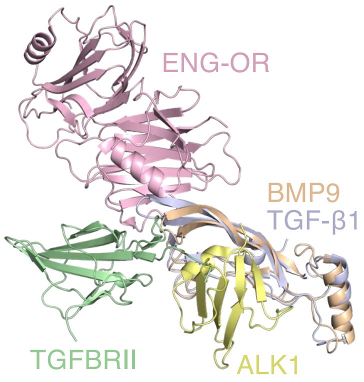

Supplementary Figure 10. Structural analysis showing ENG, TGFBRII and ALK1 can interact with BMP9 without clash. Superposition of the ENG-OR:BMP9 (5HZW) structure, the TGF-β1:TGFBRII (3KFD) structure, and the ALK1:BMP9:ActRIIB (4FAO) structure by the ligands, showing ENG, TGFBRII and ALK1 can contact BMP9 simultaneously without any clash. Figure prepared using Pymol (The PyMOL Molecular Graphics System, Version 2.0, Schrödinger).

Supplementary Figure 11

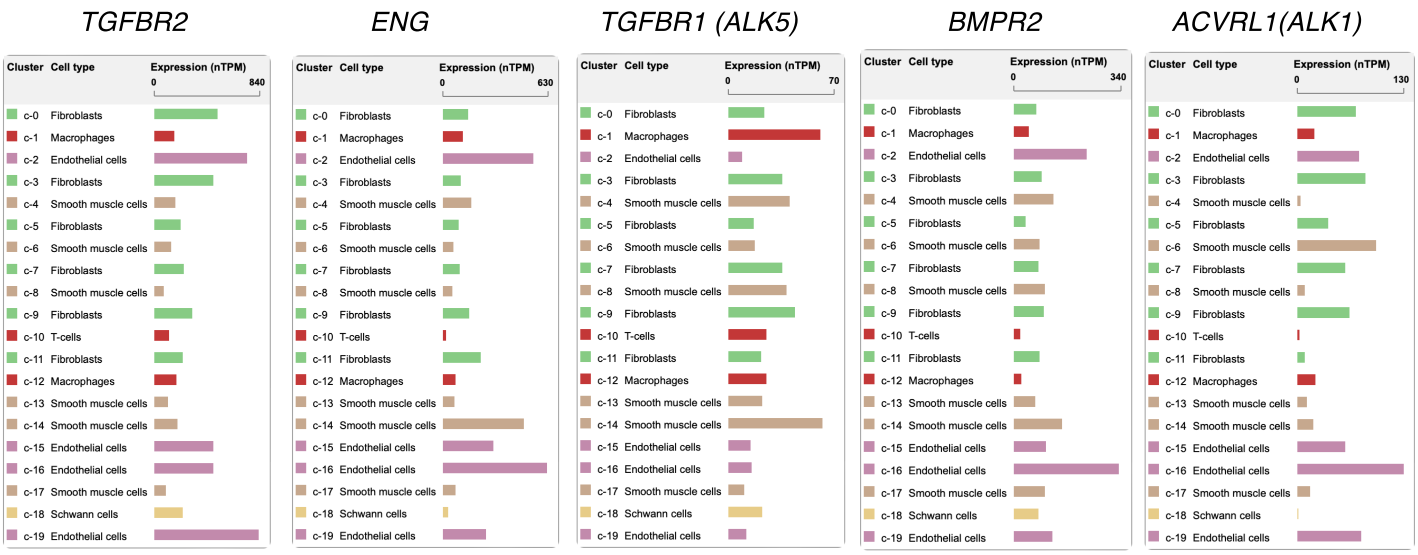

Supplementary Figure 11. *TGFBR2* is highly expressed in vascular endothelial cells. Expression levels of *TGFBR2*, *ENG*, *TGFBR1*, *BMPR2* and *ACVRL1* from single cell RNAseq datasets in vascular cells. Screenshots from the Human Protein Atlas.

(https://www.proteinatlas.org/ENSG00000163513-TGFBR2/single+cell/vascular)

IV. Supplementary References

1 Guo, J. *et al.* Crystal structures of BMPRII extracellular domain in binary and ternary receptor complexes with BMP10. *Nat Commun* **13**, 2395 (2022). https://doi.org:10.1038/s41467-022-30111-2

2 Saito, T. *et al.* Structural Basis of the Human Endoglin-BMP9 Interaction: Insights into BMP Signaling and HHT1. *Cell Rep* **19**, 1917-1928 (2017). https://doi.org:10.1016/j.celrep.2017.05.011

3 Radaev, S. *et al.* Ternary complex of transforming growth factor-beta1 reveals isoform-specific ligand recognition and receptor recruitment in the superfamily. *J. Biol. Chem.* **285**, 14806-14814 (2010). https://doi.org:10.1074/jbc.M109.079921

4 Townson, S. A. *et al.* Specificity and structure of a high affinity activin receptor-like kinase 1 (ALK1) signaling complex. *J. Biol. Chem.* **287**, 27313-27325 (2012). https://doi.org:10.1074/jbc.M112.377960
