# Supplementary material for: Endoglin as a BMP9 Co-Receptor in Vascular Endothelial Cells: Prodomain Displacement and TGFBRII Recruitment
Source: Nat Commun. Author manuscript; Available in PMC 2026 Feb 14. (PMC12824264; doi:10.1038/s41467-025-67531-9)
Supplement: Supplementary Information [file EMS211989-supplement-Supplementary_Information.zip › 41467_2025_67531_MOESM5_ESM.pdf]

nature portfolio

Corresponding author(s): Wei Li

Last updated by author(s): Nov 14, 2025

Reporting Summary

Nature Portfolio wishes to improve the reproducibility of the work that we publish. This form provides structure for consistency and transparency in reporting. For further information on Nature Portfolio policies, see our Editorial Policies and the Editorial Policy Checklist.

Statistics

For all statistical analyses, confirm that the following items are present in the figure legend, table legend, main text, or Methods section.

n/a	Confirmed	
☐	☒	The exact sample size (*n*) for each experimental group/condition, given as a discrete number and unit of measurement
☐	☒	A statement on whether measurements were taken from distinct samples or whether the same sample was measured repeatedly
☐	☒	The statistical test(s) used AND whether they are one- or two-sided<br>*Only common tests should be described solely by name; describe more complex techniques in the Methods section.*
☒	☐	A description of all covariates tested
☐	☒	A description of any assumptions or corrections, such as tests of normality and adjustment for multiple comparisons
☐	☒	A full description of the statistical parameters including central tendency (e.g. means) or other basic estimates (e.g. regression coefficient) AND variation (e.g. standard deviation) or associated estimates of uncertainty (e.g. confidence intervals)
☐	☒	For null hypothesis testing, the test statistic (e.g. *F*, *t*, *r*) with confidence intervals, effect sizes, degrees of freedom and *P* value noted<br>*Give P values as exact values whenever suitable.*
☒	☐	For Bayesian analysis, information on the choice of priors and Markov chain Monte Carlo settings
☒	☐	For hierarchical and complex designs, identification of the appropriate level for tests and full reporting of outcomes
☐	☒	Estimates of effect sizes (e.g. Cohen's *d*, Pearson's *r*), indicating how they were calculated

Our web collection on statistics for biologists contains articles on many of the points above.

Software and code

Policy information about availability of computer code

Data collection	Bulk RNAseq was conducted by Novogene (www.novogene.com) using the Novogene NGS Stranded RNA Library Prep Set (PT044). RNA sequencing was carried out by Novogene using an Illumina NovaSeq X Plus system to generate 150 bp paired end reads.
Data analysis	Raw data processing and quality control were performed by Novogen using in-house perl scripts. The reads were aligned to the mus musculus GRCm39 reference genome using Hisat2(v2.0.5). Mapped reads and sample metadata were imported into R (v.4.0.3) for downstream analysis. First, BiomaRt (v.2.54.0) mapped the ensemble gene IDs to HGNC symbols. After performing quantile normalisation, COMBAT from the sva package was applied to remove batch effects. Differential expression analysis was conducted using DESeq2 (v.1.26.0) comparing the different sample groups. The Benjamini-Hochberg method adjusted the raw p-values for the False Discovery Rate (FDR). Pathway analysis was assessed using String server (https://string-db.org/). References for the softwares are provided in the methods part of the manuscript. The code for survival analysis in R has been deposited on GitHub (and linked in the methods).

For manuscripts utilizing custom algorithms or software that are central to the research but not yet described in published literature, software must be made available to editors and reviewers. We strongly encourage code deposition in a community repository (e.g. GitHub). See the Nature Portfolio guidelines for submitting code & software for further information.

Data

Policy information about availability of data

All manuscripts must include a data availability statement. This statement should provide the following information, where applicable:

- Accession codes, unique identifiers, or web links for publicly available datasets
- A description of any restrictions on data availability
- For clinical datasets or third party data, please ensure that the statement adheres to our policy

The RNAseq dataset has been deposited at the NCBI Gene Expression Omnibus with accession number GSE289309.

Research involving human participants, their data, or biological material

Policy information about studies with human participants or human data. See also policy information about sex, gender (identity/presentation), and sexual orientation and race, ethnicity and racism.

Reporting on sex and gender	We have re-analysed published human datasets. Both male and female samples were included in the original cited studies.
Reporting on race, ethnicity, or other socially relevant groupings	We have re-analysed published human datasets. Race, ethnicity or other socially relevant groupings are not relevant in our study.
Population characteristics	We have re-analysed published human datasets to validate the genes from our mechanistic study. Population characteristics is not relevant here.
Recruitment	We did not undertake any recruitment for our work.
Ethics oversight	We have re-analysed published human lung microarray datasets which did not require ethics oversight. For whole blood RNAseq and proteomic analysis, the clinical data were obtained from the National Cohort Study of Idiopathic and Heritable PAH (UK cohort; NCT01907295, East of England Ethics Committee: 13/EE/0203) with a group 1 PH diagnosis.

Note that full information on the approval of the study protocol must also be provided in the manuscript.

Field-specific reporting

Please select the one below that is the best fit for your research. If you are not sure, read the appropriate sections before making your selection.

☒ Life sciences ☐ Behavioural & social sciences ☐ Ecological, evolutionary & environmental sciences

For a reference copy of the document with all sections, see nature.com/documents/nr-reporting-summary-flat.pdf

Life sciences study design

All studies must disclose on these points even when the disclosure is negative.

Sample size	No sample-size calculations were performed. Sample size was determined to be adequate based on the magnitude and consistency of measurable differences between groups.
Data exclusions	no data exclusion in this manuscript
Replication	All replicate experiments were successful
Randomization	no randomisation is required in this manuscript
Blinding	The RNAseq data analysis was performed by the researcher who were blinded to the hypothesis of the experiment.

Reporting for specific materials, systems and methods

We require information from authors about some types of materials, experimental systems and methods used in many studies. Here, indicate whether each material, system or method listed is relevant to your study. If you are not sure if a list item applies to your research, read the appropriate section before selecting a response.

Materials & experimental systems

n/a	Involved in the study
☐	☒ Antibodies
☐	☒ Eukaryotic cell lines
☒	☐ Palaeontology and archaeology
☐	☒ Animals and other organisms
☐	☒ Clinical data
☒	☐ Dual use research of concern
☒	☐ Plants

Methods

n/a	Involved in the study
☒	☐ ChIP-seq
☒	☐ Flow cytometry
☒	☐ MRI-based neuroimaging

Antibodies

Antibodies used

Anti-phospho-Smad1/5 (Ser463/465)(41D10) antibody (9516S), Cell Signaling Technology
Anti-Smad1 antibody (9743S), Cell Signaling Technology
Anti-human CD105 antibody (555690), BD Biosciences
Anti-CD31 (553370), BD Biosciences
Anti-VE-Cadherin (555289), BD Biosciences
Anti-mouse CD102 (553326), BD Biosciences
Anti-BMP9 antibidy (BAF3209), Biotechne
Anti-mouse ENG (AF1320) Biotechne
Anti-β-actin (A5411), Sigma-Aldrich
Anti-mouse ENG (14-1051-82), Affymetrix eBioscience/Thermo Fisher Scientific
Anti-Rat Alexa Fluor 488 (A-21208), Thermo Fisher Scientific
Anti-Rat Alexa Fluor 568 (A78946), Thermo Fisher Scientific

Validation

Anti-phospho-Smad1/5 (9516S), species cross-reactivity is determined by western blot, specificity/sensitivity is determined by flow cytometric analysis. Details can be found at company website.
Anti-Smad1 antibody (9743S), validated by the company and has 219 citations as listed on their website.
Anti-human CD105 (555690), characterised and validated by the company, and have been used in previous publications as listed on the product datasheet.
Anti-CD31 (553370), validated by the company and has 983 citations as listed on their website.
Anti-VE-Cadherin (555289), validated by the company and has 182 citations as listed on their website.
Anti-mouse CD102 (553326), validated by the company and has 108 citations as listed on their website.
Anti-BMP9 antibody (BAF3209), validated and used multiple times in our previous publications (PMIDs: 33320799, 31661308, 31431534).
Anti-mouse ENG (AF1320), validated and used in our previous publication (PMID: 31431534), 74 citations on product website.
Anti-β-actin (A5441), validated by the company.
Anti-mouse ENG (14-1051-82), has been validated by the company with 25 published figures and 43 references (https://www.thermofisher.com/antibody/product/CD105-Endoglin-Antibody-clone-MJ7-18-Monoclonal/14-1051-82).
Anti-Rat Alexa Fluor 488 (A-21208), validated by the company and has 66 published figures and 1767 references.
Anti-Rat Alexa Fluor 568 (A78946), validated by the company and has 28 references.

Eukaryotic cell lines

Policy information about cell lines and Sex and Gender in Research

Cell line source(s)

human pulmonary artery endothelial cells (hPAECs), human aortic artery endothelial cells (HAOECs), human umbilical vein endothelial cells (HUVECs) and human dermal microvascular cells (HDMECs) were purchased from Lonza. Mouse lung endothelial cells (MLECs) were isolated in this study.

Authentication

Those human primary endothelial cells were authenticated by Lonza. MLECs isolated from current study were stained with CD31, VE-cadherin and endoglin to validate the endothelial cell identity.

Mycoplasma contamination

All cells tested negative for mycoplasma

Commonly misidentified lines
(See ICLAC register)

No cell lines used are listed in the database of commonly misidentified cell lines.

Animals and other research organisms

Policy information about studies involving animals; ARRIVE guidelines recommended for reporting animal research, and Sex and Gender in Research

Laboratory animals

Engfl/fl;Rosa26-CreERT2 carrying the Immorto mouse transgene were used for isolating the endothelial cells for further in vitro studies. This mouse line has been described previously (PMID: 31805812, PMID: 31431534, PMID: 20224041, PMID: 23401487)

Wild animals

no wild animals used in this study

Reporting on sex	We did not observe any difference in the cells isolated from male or female mice, hence this was not reported.
Field-collected samples	no field-collected samples in this study
Ethics oversight	All procedures were approved by the University of Cambridge Animal Welfare Ethical Review Board under the authority of the United Kingdom Home Office Project Licences PP7550697 and PP3923706.

Note that full information on the approval of the study protocol must also be provided in the manuscript.

Clinical data

Policy information about clinical studies

All manuscripts should comply with the ICMJE guidelines for publication of clinical research and a completed CONSORT checklist must be included with all submissions.

Clinical trial registration	NCT01907295
Study protocol	The clinical data were obtained from the National Cohort Study of Idiopathic and Heritable PAH (UK cohort; NCT01907295, East of England Ethics Committee: 13/EE/0203) with a group 1 PH diagnosis. The analyses in this manuscript (Figure 9e) included patients with IPAH and HPAH diagnosed at 18 years of age or older.
Data collection	Clinical data was collected prospectively at annual outpatient visits and the last study census took place on 01/07/2022. If patients were diagnosed before the study start date, diagnostic parameters were included retrospectively in the study.
Outcomes	long term survival data is collected as part of the cohort study

Plants

Seed stocks	*Report on the source of all seed stocks or other plant material used. If applicable, state the seed stock centre and catalogue number. If plant specimens were collected from the field, describe the collection location, date and sampling procedures.*
Novel plant genotypes	*Describe the methods by which all novel plant genotypes were produced. This includes those generated by transgenic approaches, gene editing, chemical/radiation-based mutagenesis and hybridization. For transgenic lines, describe the transformation method, the number of independent lines analyzed and the generation upon which experiments were performed. For gene-edited lines, describe the editor used, the endogenous sequence targeted for editing, the targeting guide RNA sequence (if applicable) and how the editor was applied.*
Authentication	*Describe any authentication procedures for each seed stock used or novel genotype generated. Describe any experiments used to assess the effect of a mutation and, where applicable, how potential secondary effects (e.g. second site T-DNA insertions, mosiacism, off-target gene editing) were examined.*
